# Histone modification of endothelial-mesenchymal transition in cardiovascular diseases

**DOI:** 10.3389/fcvm.2022.1022988

**Published:** 2022-12-07

**Authors:** Qiu Jun, Li Youhong, Zhong Yuan, Yang Xi, Bingyu Wang, Sun Xinyi, Yin Fu, Cen Kedan, Jiangfang Lian, Zhou Jianqing

**Affiliations:** ^1^Li Huili Hospital Affiliated to Ningbo University, Ningbo, China; ^2^Medicine School of Ningbo University, Ningbo, China; ^3^Ningbo Medical-Industrial Integration Innovation Research Institute, Ningbo, China

**Keywords:** atherosclerosis, EndMT, histone methylation, histone acetylation, HDACs

## Abstract

Endothelial-mesenchymal transition (EndMT) is a differentiation process in which endothelial cells lose their own characteristics and acquire mesenchymal-like characteristics, which contributes to the formation and development of atherosclerotic plaques. Until now, there is still a lack of effective measures to treat atherosclerosis (AS), so there is an urgent need to understand the underlying mechanisms of AS. In addition, although various studies have shown that EndMT is involved in the pathological stages of cardiovascular diseases, such as myocardial fibrosis, myocardial hypertrophy, and hypertension, the specific molecular mechanisms driving EndMT are still in the exploratory stage. In this review, we review the role of histone modifications (methylation, demethylation and acetylation, deacetylation) on EndMT in cardiovascular disease, aiming to target histone-modifying enzymes to guide cardiovascular disease therapy.

## Introduction

Endothelial-mesenchymal transition (EndMT) is a special state of endothelial cells to mesenchymal cells, and the cell morphology changes from oval to spindle-like fibroblasts, with the loss of intercellular connections and polarity, migration and collagen synthesis capacity increases. When endothelial cells undergo EndMT, endothelial cell-specific markers such as CD31, VE-cadherin, and eNOS are lost, and mesenchymal markers α-SMA, SM22α, and Vimentin are gained (1). Originally EndMT was discovered during heart development and involved in the formation of heart valves and interventricular septum (2). In 2007, it was first discovered in adult organisms that EndMT can promote myocardial fibrosis and participate in the process of pathological tissue remodeling (3). In recent years, researches on EndMT and atherosclerosis (AS), pulmonary hypertension, myocardial fibrosis and other cardiovascular diseases have been carried out continuously, and it has been suggested that EndMT is the main contributor to these diseases (4–6). Therefore, targeting EndMT may be of great significance in the prevention and treatment of cardiovascular diseases. However, EndMT is a process influenced by complex environmental factors, and its contribution to cardiovascular events varies across studies, therefore, we still need to further explore the different environmental factors and signaling pathways that affect EndMT and related mechanisms.

## Main regulatory pathways and influencing factors of endothelial-mesenchymal transition

Transforming growth factor-β (TGF-β) is a major inducer of EndMT and regulates vascular homeostasis (7). There are three family members: TGF-β1, TGF-β2, and TGF-β3, of which TGF-β2 is the strongest inducer of EndMT, while TGF-β1 and TGF-β3 can induce EndMT process through paracrine TGF-β2 (8). TGF-β signaling is induced by ligands and serine/threonine protein kinases. The ligand first binds to the cognate cell membrane type II receptor, phosphorylates and activates type I receptors (ALK1 and ALK5), and then Smads (R- Smads) phosphorylation, the activated Smads and Smad4 assemble into a complex into the nucleus, where the transcription factors Snail, Twist, and Slug are activated to regulate the EndT process (9, 10). The effect of TGF-β on EndMT depends on the activated TGF-β type I receptors. ALK5 promotes EndMT through Smad2/3 signaling pathway, while ALK1 activates Smad1/5 and antagonizes EndMT by antagonizing ALK5 (11).

**FIGURE 1:**
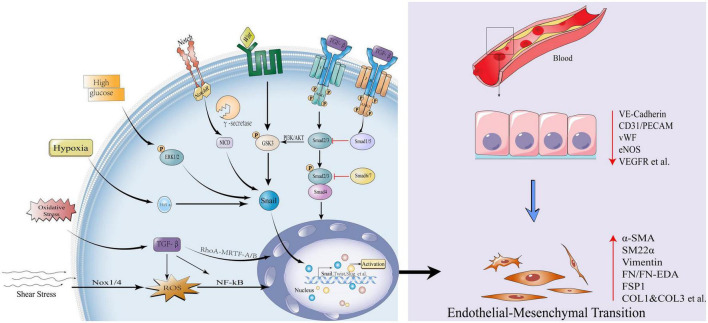
Influencing factors and canonical signaling pathways of endothelial-mesenchymal transition. Transforming growth factor-β (TGF-β) signal is initiated, type II receptor activates type I receptor ALK1, phosphorylates Smad2/3, and the latter combines with Smad4 to form a complex into the nucleus to activate transcription factors Snail, Twist1, Slug et al., promoting EndMT; While the type II receptor activates the type I receptor ALK5 and inhibits EndMT *via* Smad1/5; Smad6/7 also inhibits EndMT by Smad2/3; The non-Smad pathways activated by TGF-β include: PI3K/Akt, Rho GTPase, etc. Other pathways that activate EndMT: Wnt/β-catenin and Notch signaling pathways. High glucose, hypoxia, oxidative stress, and shear stress can all activate the EndMT process through various mechanisms. EndMT manifests as loss of endothelial markers: Vascular endothelial cell-cadherin, VE-cadherin, Platelet endothelial cell adhesion molecule-1, CD31/PECAM, von Willebrand Factor, vWF, endothelial Nitric Oxide Synthase, eNOS, Vascular endothelial growth factor receptor, VEGFR; Obtainment of mesenchymal markers: Actin alpha 2,α-SMA/ACTA2, Smooth muscle 22α, SM22α, Vimentin, Fibronectin, FN/FN-EDA, Fibroblast-specific protein 1, FSP1, Collagen Type I and Collagen Type 3, COL1 and COL3 etc.

In addition, TGF-β can also affect the EndMT process by regulating non-Smad pathways, such as ERK/MAPK, PI3K/Akt (12) and Rho GTPase (13). Other regulation of EndMT signaling pathways, such as Wnt/β-catenin, Notch and Hippo pathways can participate in EndMT process alone or in conjunction with TGF-β (14–16). Under pathological conditions (17, 18), Wnt/β-catenin signal can induce EndMT by inhibiting glycogen synthase kinase 3β (GSK3β)-mediated phosphorylation. This is partly due to the fact that GSK-3β is a major kinase that phosphorylates Snail and induces the degradation of Snail (19). TGF-β2 can activate AKT through the PI3K pathway, which can phosphorylate GSK-3β, stabilize Snail protein, and promote EndMT (20). In addition, systemic risk factors, such as hyperglycemia (21), hypoxia (22), blood flow shear stress (23), oxidative stress (13), etc., also regulate the EndMT process and affect the progression of cardiovascular disease (Figure 1). Although these signaling pathways and environmental factors affect the occurrence and development of EndMT, the specific regulatory mechanisms need to be further explored. With the in-depth study of epigenetics, numerous evidences have shown that various histone modifications and non-coding RNAs are involved in the EndMT process, and play an important role in the occurrence and development of cardiovascular diseases.

## Epigenetic modification and endothelial-mesenchymal transition

Epigenetic modification is a bridge connecting genetic and environmental factors. It does not modify the DNA sequence, but can regulate how genes are expressed, including DNA methylation, histone modification, chromatin remodeling, non-coding RNA editing and other gene regulation methods. The dysregulation of any link will affect the abnormality of chromatin structure and gene expression, which will lead to the occurrence of diseases (24, 25). In recent years, studies have found that epigenetic modification plays an important role in the occurrence and development of cardiovascular diseases, and many epigenetic changes are reversible, which provides an optimistic prospect for disease treatment (26, 27). Because EndMT is involved in cell development, it is a state of cell differentiation, and is involved in epigenetic regulation-related diseases, therefore, exploring the role of different epigenetic modifications in the mechanism of EndMT may help us to combat EndMT-related pathologies, and then guide the treatment of cardiovascular diseases and the development of new drugs.

## Histone modifications regulate endothelial-mesenchymal transition

Histone is a basic protein in eukaryotic cell chromatin and prokaryotic cells, histone H2A, H2B, H3, and H4 are surrounded by DNA to form nucleosome structure. The tails of histones are targets of epigenetic modifications that can regulate gene expression. Histones can alter gene transcription in several ways: methylation, acetylation, phosphorylation, ubiquitination, SUMOylation, etc. (28). Histone methylation modification is regulated by histone methyltransferases (HMTs) and histone demethylases (HDMs). According to different methylation sites and states, methylated histones can be activated (H3K4me2/3, H3K36me1/3, H3K79me1/2, and H4K20me1) or inhibit (H3K9me2/3, H3K27me2/3, H3K79me3, and H4K20me3) gene transcription (29). Similarly, another common acetylation modification is acetyltransferases (HATs) and deacetyltransferases (HDACs) that turn gene transcription on and off. Under the action of different enzymes, histones start the process of methylation modification and acetylation modification. The dynamic balance between different enzymes ensures the orderly expression of genes and the balance of various functions of the body (30). The regulation of these enzymes has made initial progress at the molecular level and clinical, this article focuses on the research on HMTs, HDMs, HATs, HDACs, and the regulation of EndMT and the underlying mechanism, aiming to explore possible targets for the treatment of cardiovascular diseases from an epigenetic perspective.

## Histone methyltransferase enhancer of zeste homolog 2 and demethylase JMJD2B regulate endothelial-mesenchymal transition in cardiovascular disease

The main cause of most cardiovascular diseases is AS. As is a chronically progressive inflammatory disease in which lipid deposition, hemorrhage, and thrombosis occur in diseased arteries, followed by fibrosis and calcification. This eventually leads to a hardening of the arterial wall and a narrowing of the lumen (31). Many studies have confirmed the importance of fluid shear stress in mediating EndMT and AS, but the specific molecular mechanism still needs to be explored. Recently, epigenetic mechanisms have attracted attention in the regulation of flow shear stress (32–34). Enhancer of zeste homolog 2 (EZH2) is the major methyltransferase of Polycomb repressive complex 2 (PRC2), its SET domain catalyzes the trimethylation of histone H3 lysine 27 (H3K27me3), thereby maintaining the silencing state of downstream target genes (35, 36). EZH2 plays an important role in endothelial cell dysfunction. In blood flow low shear stress or oscillatory shear stress (atherosclerosis susceptible area), endothelial protective mitogen activated protein kinase 7 (MAPK7) signaling pathway is inhibited to promote EndMT (37), the increased expression of EZH2, which further promotes endothelial cell hyperproliferation, leads to endothelial cell dysfunction (38). During High Uniform laminar shear stress (atherosclerotic protection zone), activated MAPK7 reduced EZH2 expression by increasing the EZH2 inhibitor microRNA-101. In turn, decreased EZH2 promotes MAPK7 activation by reducing the phosphatase activity responsible for MAPK7 inactivation. If the balance between the two is broken, it will induce EndMT and lead to cardiovascular disease (38, 39). In addition to hemodynamic abnormalities, EZH2 regulates EndMT in other microenvironments. Left atrium isolated from mice treated with Ang-II, showing high levels of EZH2 expression and H3K27me3 methyltransferase activity. EZH2 forms a transcriptional complex with Smad2 under the action of Ang-II, mediates the activation of α-SMA gene, and promotes fibroblast differentiation. α-SMA, as a mesenchymal gene, is up-regulated in EndMT, thereby promoting EndMT. This process was inhibited by the EZH2 inhibitor GSK126 and reversed the differentiation of atrial fibroblasts in patients with sinus rhythm and atrial fibrillation (40). However, in the context of endothelial cells under the action of TGF-β and IL-1β, inhibition of EZH2 attenuated the inhibitory effect of H3K27me3 on the TAGLN promoter and activated the transcription of the mesenchymal gene TAGLN/SM22α, so that EZH2 negatively regulates EndMT (41). Therefore, the exact role of EZH2 on EndMT remains controversial. A recent study found that the EZH2 inhibitor GSK126 successfully reduced atherosclerotic plaque progression in ApoE-/- mice (42).

**FIGURE 2:**
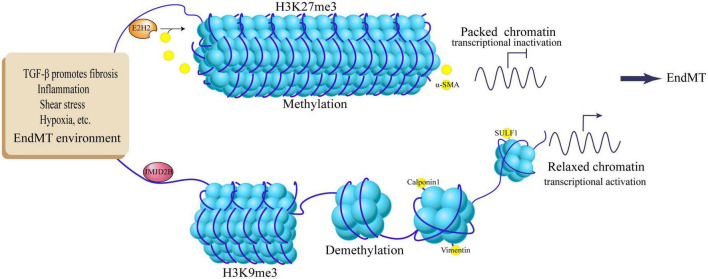
Regulation of EndMT by histone methylation and demethylation. TGF-β promotes fibrosis, inflammation, shear stress, hypoxia and other EndMT environments, up-regulates JMJD2B expression, demethylates histone H3K9me3, activates target genes Calpoin1, Vimentin, SULF1, and promotes EndMT process; EndMT stimulators activate E2H2, methylate histone H3K27me3, inhibit the expression of target gene α-SMA, and inhibit the EndMT process.

The extracellular matrix protein sulfatase 1 (SULF1) is a heparan sulfate proteoglycan involved in epithelial cell migration (43), which regulates two key EndMT pathways: promoting TGF-β signaling and post-transcriptionally inhibiting FGF signaling pathway (44, 45). This key regulator is epigenetically controlled by the HDM JMJD2B, which increases SULF1 expression by reducing the H3K9me3 mark, thereby activating TGF-β signaling and promoting the EndMT process (46). Small interfering RNA silencing of JMJD2B attenuates TGF-β-induced expression of NAPDH oxidase 4 (NOX4) and reduces intracellular reactive oxygen species (ROS) levels, which partially reverses the EndMT process in tissue fibrosis (47). On the other hand, JMJD2B can enhance β-catenin nuclear localization and transcription, and bind to the promoter of the β-catenin target gene Vimentin to increase its transcriptional activity by inducing H3K9 demethylation, thereby promoting EndMT (48). In addition, JMJD2B is regulated by a variety of environmental factors, Under the action of EndMT contributing factors such as hypoxia, disturbed flow, and inflammatory stimulation, up-regulation of JMJD2B expression can demethylate H3K9me3, thereby reducing the inhibitory effect of H3K9me3 on the mesenchymal marker gene Calponin1 (CNN1). At the molecular level, the JMJD2B promoter has a conserved hypoxia response element that binds to HIF-1α (49). It indicates that JMJD2B may be an important regulator of endothelial cell phenotype, and it cooperates with TGF-β signaling and Wnt/β-catenin signaling to positively regulate the EndMT process under the participation of multiple factors. Recent studies have found that JMJD2B can inhibit the expression of lncRNA-AERRIE during EndMT, and lncRNA-AERRIE in turn regulates SULF1, causing changes in EndMT markers (50). However, lncRNA-AERRIE can promote lymphoma EMT process by activating Snail (51), deletion or overexpression of lncRNA-AERRIE itself did not affect the EndMT process, so it would be interesting to explore the association of JMJD2B with lncRNA-AERRIE and SULF1 in the context of EndMT (Figure 2).

## Histone acetyltransferase p300 regulate endothelial- mesenchymal transition

The study found that fibroblast-like cells can be derived from endothelial cells through EndMT, which can produce a large amount of collagen and extracellular matrix to participate in the process of cardiac fibrosis. In EndMT-derived fibroblast-like cells, transcript and protein levels of α-SMA, Snail, β-catenin, and acetyltransferase p300 were elevated, suggesting that elevated p300 levels may contribute to EndMT (52). The use of p300 small molecule inhibitors-L002 and C646 can specifically inhibit the activity of p300 acetyltransferase and block the differentiation of fibroblasts to myofibroblasts induced by TGF-β. They can attenuate p300-mediated specific histone acetylation (H4, H3K9) to inhibit collagen transcription, not only control the occurrence of EndMT, but also reverse hypertension-induced cardiac hypertrophy and fibrosis (53). These studies suggest that p300 is involved in the regulation of EndMT.

p300 can be regulated under the conditions of different EndMT stimulators, and its mediated acetylation modification can further regulate the EndMT process. TGF-β is one of the strongest stimulators of EndMT, and TGF-β1 stimulates the phosphorylation of Smad2/3, which directly interacts with p300/CBP in the nucleus and upregulates the HAT activity of p300 (54). In turn, elevated p300 leads to chromatin acetylation of target gene promoters, promotes transcriptional activation of Smad2/3, and enhances TGF-β signaling (55). Likewise, Friend leukemia integration-1 (Fli1), a potent inhibitor of collagen gene expression in dermal fibroblasts, is also a known target of p300. TGF-β1 induces acetylation of Fli1 by activating the p300 HAT domain, thereby reducing its stability. Downregulation of Fli1 expression can drive the EndMT program, which plays an important role in scleroderma skin fibrosis (56, 57). In addition, high glucose (25 mmol/L) induces endothelial cell damage and an inflammatory phenotype, which in turn triggers EndMT. Exposure to high glucose enhances the activity of the transcriptional co-regulator p300, which increases the activity of TGF-β through Smad2 acetylation, which further aggravates endothelial cell damage and the EndMT process. Using curcumin, a known p300 inhibitor, *in vitro* and *in vivo*, Antoinette et al. (58) found that inhibition of p300 activity can reduce Smad acetylation and TGF-β activity, and reduce cardiac hypertrophy, improve diastolic function, and reduce extracellular matrix production, but not affect blood sugar. Similarly, curcumin has been shown to inhibit IL-6-dependent EndMT and attenuate renal transplant fibrosis through autophagy activation (59).

Dennis, and colleagues (60) used Immunoprecipitation-western blot to find that p300 interacts with the EndMT-specific transcription factor Snail and acetylates Snail at lysine 146 (K146) and K187, thereby reducing Snail ubiquitination and enhancing its protein stability. p300 is a mediator of Wnt transcriptional activity, and its mediated Wnt pathway transcriptional activity is related to cell differentiation. In addition to affecting the effects of butyrate on Wnt signaling, apoptosis and proliferation to varying degrees, P300 can also bind to the Wnt signaling target gene ZEB1 to enhance ZEB1 transcription and promote the EndMT process (61). p300 can also activate HIF-1 (positive regulator of VEGF and Twist) and β-catenin, which then induce EndMT-like phenotypic changes (62). All in all, as an important epigenetic regulator, p300’s regulatory role in EndMT cannot be ignored, and p300-targeted therapy has made initial progress in combating EndMT.

## Regulation of endothelial- mesenchymal transition by histone deacetylases in cardiovascular disease

Various studies have shown that in HDAC1-10 of human ruptured atherosclerotic lesions, HDAC3 is the only gene that up-regulates HDAC, especially the AS susceptible area [inflammation area (63) and blood flow disturbance area (64)]. The specific knockout of HDAC3 in mouse macrophages can promote collagen deposition in atherosclerotic lesions and reduce lipid accumulation (63). Previous studies have proved that HDAC3 undergoes unconventional splicing in stem cell differentiation to produce HD3α isoform, which induces the EndMT phenotype in mature endothelial cells (65). The use of HDAC3 specific inhibitor RGFP966 can inhibit EndMT by reducing the inflammatory response in ApoE-/-mouse AS (66). HDAC4 knockdown leads to inhibition of Ang II-mediated α-SMA expression and ERK phosphorylation, and inhibits the process of myofibroblast transdifferentiation (67). HDAC2 (68) and HDAC8 (69) can activate a variety of EndMT and pro-fibrotic signals and transcription factors, inhibit anti-fibrotic proteins, and promote the (70)process of EndMT and fibrosis. In addition, Class IIa HDAC inhibition and endothelial-specific HDAC9 knockout both reverse the phenotypic changes of endothelial cells induced by EndMT, resulting in a decrease in lipid content in the plaque and an increase in the thickness of the fibrous cap, leading to a more favorable plaque phenotype (71). Therefore, targeting HDACs to improve EndMT and then treat AS will have a good prospect. The existing HDACI targeting EndMT will be described later.

SIRTs are a type of NAD + dependent histone deacetylases that are widely present in life. They are involved in inflammation (72), oxidative stress (73), aging (74) and other activities. These activities are related to endothelial cell dysfunction and cardiac dysfunction, vascular diseases are closely related (75). The expression of SIRT1 is down-regulated in EndMT induced by TGF-β, and the activation of SIRT1 can inhibit EndMT induced by TGF-β. The mechanism involved may be that SIRT1-mediated Smad7 deacetylation can enhance Smad ubiquitination regulator 1 (Smurf1)-mediated degradation of ubiquitin proteasome, resulting in a decrease in Smad7 expression (76). On the other hand, SIRT1 can directly deacetylate Smad4 to inhibit EndMT of endothelial cells induced by TGF-β (77). The occurrence of pathological EndMT in AS plaques requires activation of the MALAT1-dependent Wnt/β-catenin signaling pathway (78). SIRT6 can directly bind to the LncRNA MALAT1 promoter and inhibit its expression in ECs (79). In short, targeting specific HDACs can interfere with abnormal EndMT signals and provide a new way to combat EndMT.

**FIGURE 3:**
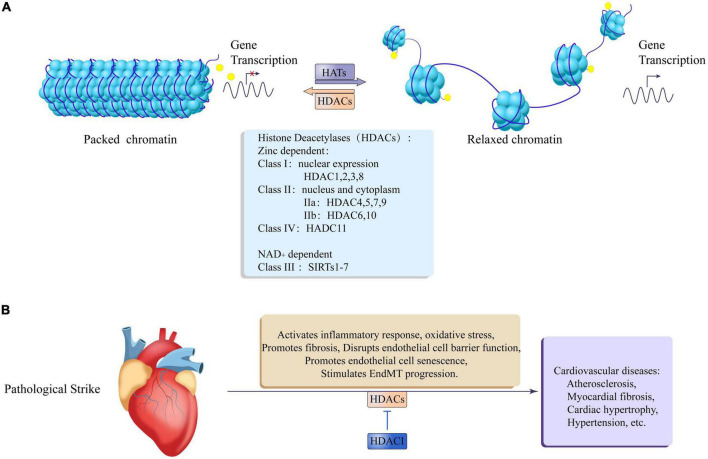
Epigenetic regulation of histone acetylation and deacetylation in cardiovascular disease. **(A)** Histone acetyltransferases (HATs) relax packaged chromatin to promote gene transcription, and histone deacetylases (HDACs) promote chromatin packaging to inhibit gene transcription. The HDACs family is divided into zinc lipoprotein dependent and NAD + dependent, Zinc lipoprotein dependent enzymes include: class I: HDAC1, 2, 3, 8, which are mainly expressed in the nucleus; class II: IIa: HDAC4, 5, 7, 9; IIb: HDAC6, 10, which are expressed in both the nucleus, nucleus, and cytoplasm; class IV: HDAC11. NAD + dependent enzymes include: Class III: SIRTs1-7. **(B)** After the heart is pathologically stimulated, HDACs can activate an inflammatory response and oxidative stress, promote fibrosis, damage endothelial cell barrier function, and promote endothelial cell senescence and EndMT process leading to cardiovascular diseases such as atherosclerosis, myocardial fiber Cardiac hypertrophy, hypertension, etc. At the same time, HDACI Can antagonize the adverse effects of HDACs.

SIRTs have been proven to maintain endothelial homeostasis through their antioxidant effects (80). Various studies have shown that excessive production of ROS can induce EndMT, damage endothelial cell function, and then affect the progression of AS plaques (81, 82). SIRT3 activates the expression of catalase by inducing forkhead box O3a (Foxo3a) deacetylation and nuclear localization, thereby reducing oxidative stress and inhibiting the EndMT process. In SIRT3 knockout mice, AngII-induced EndMT and renal fibrosis can be aggravated, while in endothelial-specific transgenic SIRT3 mice, Ang II-induced EndMT and renal dysfunction can be improved (83). Interestingly, SIRT3-/- did not change blood pressure. In hypertensive kidney injury, it may be that the lack of SIRT3 can impair the systolic function of the heart (84). Similarly, inhibiting SIRT1 activity can enhance the expression of fibronectin and TGF-β1 induced by the advanced glycation end product (AGE). Overexpression of resveratrol or SIRT1 can prevent this effect, thereby inhibiting mesenchymal transition (85). Obviously, the regulation of EndMT by HDACs is not limited to these examples, and the HDACs related to EndMT are summarized in the following Table 1 and Figure 3.

**TABLE 1:** Effects of HDACs on EndMT.

HDACs	Inhibitor	Pathways and corresponding mechanisms	Experimental model	Disease	References
**Inducing EndMT**					
HDAC1	Vaccarin	ROS/p38 MAPK signaling pathway	*In vitro* human umbilical vein endothelial cells treated with ox-LDL	Atherosclerosis	(70, 96)
HDAC2	Trichostatin A	ROS	*In vivo* streptozotocin (STZ)-induced diabetic mice; *In vitro* TGF-β1-treated rat renal tubular epithelial cells	Diabetic nephropathy	(68)
HDAC3	RGFP966	Vascular endothelial cell inflammatory response	*In vivo* ApoE-/- mice and C57/B6 mice; *In vitro* human umbilical vein endothelial cells (HUVECs)	Atherosclerosis	(66)
HDAC4	Valproic acid (VPA)	MAPK/ERK signaling pathway	*In vivo* Angiotensin II-induced myocardial fibrosis mice	Myocardial fibrosis	(67)
HDAC8	PCI34051	Activates Smad3, STAT3, β-catenin and snail	*In vivo* unilateral ureteral obstruction (UUO)-induced renal fibrosis in mice	Renal fibrosis	(69)
HDAC9	MC1568	Deacetylation of histone H3 lysine residues	*In vivo* endothelial-specific HDAC9 knockout mice; *In vitro* human coronary endothelial cells (HCAECs) and human umbilical vein endothelial cells (HUVECs)	Atherosclerosis	(70)
SIRT4	–	Antagonize Sirt3 and increase ROS accumulation	*In vivo* male C57BL/6 Sirt4 knockout mice, transgenic (Tg) mice exhibiting cardiac-specific overexpression of Sirt4 (Sirt4-Tg)	Pathological cardiac hypertrophy	(97)
SIRT6	Small interfering RNA (si-RNA)	Binds LncRNA MALAT1 promoter to inhibit Wnt/β-catenin signaling pathway	*In vivo* C57BL/6 mice; *In vitro* human aortic endothelial cells (HAECs)	Vascular aging	(79)
**Inhibiting EndMT**					
SIRT1	Short hairpin RNA (shRNA)	Decreases Smad7 expression; Deacetylated Smad4	*In vitro* human umbilical vein endothelial cells (HUAECs)	Cardiac fibrosis	(76, 77)
SIRT3	Catalytic mutant of SIRT3	SIRT3-Foxo3a-catalase pathway	*In vivo* Endothelial-specific SIRT3 knockout mice; *In vitro* primary mouse glomerular endothelial cells	Hypertensive kidney damage	(83)
SIRT7	Short hairpin RNA (shRNA)	Inhibition of DAPK3 transcription and inflammatory response	*In vivo* diabetic nephropathy patients and rats; *In vitro* glomerular endothelial cells	Diabetic nephropathy	(98)

In short, these studies have proved the existence of the pathological HDACs/EndMT/AS axis. The involvement of HDACs in EndMT-related pathways in AS needs further study. HDACs as targets may be expected to treat the progression of atherosclerotic disease and stabilize plaque.

## Regulation of histone ubiquitination by endothelial- mesenchymal transition

Ubiquitin is a small molecule protein composed of 76 amino acids, which is ubiquitinated by ubiquitination under the action of ubiquitinase. Histone ubiquitination affects gene expression by affecting chromosomal structure. Inhibition of ubiquitination has been reported in preclinical studies to prevent systemic sclerosis (86). Histone ubiquitination is also increasingly recognized in cardiovascular disease, especially during EndMT (87). Snail is one of the specific transcription factors that induce EndMT. Snail can interact with the ubiquitin E3 ligase Ring1B, and Snail’s carboxyl zinc finger recruits Ring1B and its paralog Ring1A to repress its target promoter. Deletion of Ring1A and Ring1B reduces Snail binding to target chromatin and reduces histone 2A (H2A) monoubiquitination at K119, thereby inhibiting Snail-mediated transcription and cell migration (88). In addition, TGF-β-induced EndMT is dependent on the transcription of downstream genes and crosstalk between pathways, and inhibitory Smads (Smad6/7) can recruit SMAD-specific E3 ubiquitin protein ligase 1 (SMURF2) to degrade activated TβRI through ubiquitination, thereby inhibiting the EndMT process (89). SMURF1 can inhibit TGF-β1/Smad3/4-induced vascular endothelial growth factor (VEGF) expression and reduce EndMT-involved angiogenesis process (90). Long non-coding RNA SENCR can directly inhibit SMURF2-mediated TGF-β/Smad signaling pathway and inhibit EndMT process (91). MEK/ERK acts as a TGF-β non-canonical Smad signaling pathway, and its activation can promote the production of collagen and now connexin, and promote the EndMT process. MEK ubiquitination occurs on lysine 104, which promotes MEK/ERK pathway activation and fibroblast maturation, and both Ras and GRB2 ubiquitination can promote MEK/ERK pathway activation and promote fibrosis (92).

SUMO1 is a modifier in the process of ubiquitination. In a hypoxic mouse model of pulmonary hypertension, SUMO1 expression is significantly increased, and it is involved in the process of pulmonary artery vascular smooth muscle dedifferentiation (α-SMA, SM22αreduction), that is, mesenchymal-endothelial transition process. Targeting SUMO1 not only reversed EndMT, but also treated pulmonary hypertension by inhibiting autophagic signaling (93). The sarcoplasmic/endoplasmic reticulum Ca2 + -ATPase (SERCA), a key determinant of cardiac function, whose enzymatic activity is reduced is a major feature of heart failure. Studies have found that ubiquitination is necessary to maintain the activity and stability of SERCA2a ATPase, and post-translational modification of SUMO1 can enhance the activity of SERCA2a to improve cardiac function (94), while the use of SERCA-specific inhibitor thapsigargin can induce oxidation Stress aggravates the EndMT process, which leads to the development of fibrosis (95). The role of other epigenetic regulation in SERCA2a activity has also been demonstrated, with acetylation of lysine 492 significantly reducing SERCA2a activity by interfering with ATP binding to SERCA2a in both human and animal models of heart failure. Acetylation/deacetylation of lysine 492 is mediated by P300 and SIRT1, and pharmacological activation of SIRT1 can restore SERCA2a activity and failing hearts through deacetylation of lysine 492 (94).

## Epigenetic regulation of endothelial-mesenchymal transition-specific transcription factors

Endothelial-mesenchymal transition is controlled by several key transcription factors such as Snail1, Slug (Snail2), Twist, ZEB, and KLF4. The abnormal activation of these transcription factors promotes the occurrence of EndMT, which leads to the development of a variety of cardiovascular diseases. In normal endothelial cells, these genes are usually silenced or expressed at low levels to maintain endothelial properties, but when EndMT is activated by pathways such as TGFβ, Wnt, and pro-inflammatory cytokines, these genes are activated. For example, activated Snail1 interacts with SMAD3/4 to inhibit endothelial gene expression, thereby increasing TGF-β-mediated mesenchymal transformation (96).

Dennis, and colleagues (60) used Immunoprecipitation-western blot to find that p300 can interact with EndMT-specific transcription factor Snail and acetylate Snail at lysine 146 (K146) and K187, thereby reducing Snail ubiquitination and stabilizing Snail. The acetyltransferase CREB-bindinq protein (CBP) catalyzes the interaction between its HAT domain and the C-terminal domain of Slug, acetylating Slug at positions 166 and 211 of lysine, doubling its half-life and increasing stability (97). Mutations in these lysines will reduce their ability to transactivate target genes (98), suggesting that the transcriptional activation ability of the EndMT transcription factor requires acetylation.

In physiological endothelial cells, the La ribonucleoprotein domain family member 7 (LARP7) directly interacts with the apparent inhibitory factor TRIM28 to promote the loading of its chromatin on the Slug promoter and eliminate HDAC1-mediated histone H3 acetylation and inhibits Slug transcription, thereby maintaining endothelial identity and preventing EndMT. However, when endothelial cells are subjected to EndMT-Promoting Stimuli such as TGF-β and inflammatory factors, they reduce LARP7, destroy TRIM28-HDAC1 chromatin loading chromatin loading, increase histone acetylation, and release Slug inhibition. This plays an important role in EndMT-mediated heart valve development (99). Vascular endothelium (VE)-cadherin is considered to be a calcium-dependent cell-cell adhesion protein, and its absence is a sign of EndMT (22). A recent study found that Twist was significantly expressed during the activation of EndMT by TGF-β. At the VE-cadherin promoter, H3K9 methylation was up-regulated, and H3K4/H3K56 acetylation was down-regulated. Twist forms a functional complex with H3K9 methyltransferase and HDAC to mediate the transcriptional inhibition of VE-cadherin (100). In addition, Krüppel-like factor 4 (KLF4) is a zinc-finger–containing transcription factor, which binds to the TCE site on the promoter of EndMT-related genes, including: SM22 (101), TGF-βR (102), BMP6 (103), which is important for EndMT Regulation. Under the stimulation of TGF-β1, KLF4 separates from the phosphatase and tensin homolog (PTEN)-KLF4 complex, leading to phosphorylation of KLF4, and then it recruits p300 to the self-regulated gene promoter to activate transcription. In addition, phosphorylated KLF4 enhances p300 HAT activity through the p38 MAPK pathway, and induces H3 acetylation, which leads to gene transcription (104). HDAC8 phosphorylates Snail motif 2 in the serine-rich region to increase the stability of Snail protein (105).

A variety of epigenetic pathways control the transcriptional activation and inhibition of EndMT-specific transcription factors. Targeted transcription factors have been reported to inhibit EndMT, revealing the potential mechanism of epigenetic regulation of EndMT-related transcription factors, which may be a new anti-abnormal EndMT Treatment methods provide important clues.

## Conclusion

At present, the research on epigenetic regulation of histones in the cardiovascular field is constantly expanding, and many new discoveries and new ideas have been proposed. Various histone-modifying enzymes can be inhibited by a special inhibitor, and good progress has been made in the treatment of cardiovascular diseases. However, how the epigenetic regulation of histones finely regulates various biological progress requires further study. EndMT is involved in a variety of cardiovascular diseases, especially the formation of atherosclerotic plaques. By exploring the role of histone epigenetic modification in EndMT, it is expected to provide a new direction for the treatment of cardiovascular diseases.

## Data availability statement

The original contributions presented in this study are included in the article/supplementary material, further inquiries can be directed to the corresponding authors.

## Author contributions

QJ: article writing, analysis, drawing, and form making. LY and SX: drawing and investigation. ZY and BW: drawing. YX: editing and funding. JL: supervision and writing—review and editing. ZJ: modification. All authors contributed to the article and approved the submitted version.
